# ATXN2L upregulated by epidermal growth factor promotes gastric cancer cell invasiveness and oxaliplatin resistance

**DOI:** 10.1038/s41419-019-1362-2

**Published:** 2019-02-20

**Authors:** Li Lin, Xiaoyin Li, Changqie Pan, Wanying Lin, Ruoyang Shao, Yantan Liu, Junhao Zhang, Yuhao Luo, Kai Qian, Min Shi, Jianping Bin, Yulin Liao, Wangjun Liao

**Affiliations:** 1 Department of Oncology, Nanfang Hospital, Southern Medical University, Guangzhou, China; 2 Department of Cardiology, Nanfang Hospital, Southern Medical University, Guangzhou, China

## Abstract

For gastric cancer (GC) control, metastasis and chemoresistance are the major challenges, accompanied with various stresses. Ataxin-2-like (ATXN2L) was discovered as a novel regulator of stress granules, yet its function in cancers remained unknown. Hence, we wanted to explore the functions of ATXN2L to see whether it participates in stress-related cancer malignant activities. Clinical follow-up was performed to see the impact of ATXN2L on GC patient survival. As a result, ATXN2L expression was upregulated in GC tissue and indicated adverse prognosis for overall survival and recurrence. In GC cells, ATXN2L expression was knocked down and functional experiments were performed. ATXN2L promoted GC cell migration and invasion via epithelial to mesenchymal transition, yet no influence on proliferation was detected by ATXN2L interference. When adding the chemotherapeutic agent oxaliplatin to induce stress, silencing ATXN2L sensitized GC cells to oxaliplatin. Interestingly, oxaliplatin was found to in turn promote ATXN2L expression and stress granule assembly. Then, two acquired oxaliplatin-resistant strains were generated by long-term oxaliplatin induction. The oxaliplatin-resistant strains presented with elevated ATXN2L levels, while silencing ATXN2L in the strains reversed the oxaliplatin resistance by increasing reactive oxygen species production and apoptosis. These results suggested that ATXN2L was responsible for not only intrinsic but also acquired oxaliplatin chemoresistance. Finally, ATXN2L-related signaling was screened using bioinformatic methods, and epidermal growth factor (EGF) was verified to promote ATXN2L expression via PI3K/Akt signaling activation. Blocking EGFR/ATXN2L signaling reversed GC cell oxaliplatin resistance and inhibited migration. In conclusion, ATXN2L promotes cell invasiveness and oxaliplatin resistance and can be upregulated by EGF via PI3K/Akt signaling. ATXN2L may be an indicator and therapeutic target in GC, especially for oxaliplatin-based chemotherapy.

## Introduction

Gastric cancer (GC) is one of the most universal malignant tumors globally, especially in those less-developed regions. Metastasis and chemoresistance are the two major treatment challenges for the intermediate and advanced staged GC. In clinical practice, oxaliplatin is one of the recommended agents that used in both adjuvant and palliative GC chemotherapy, the main cytotoxic effect of which is DNA synthesis inhibition. However, intrinsic or acquired resistance to oxaliplatin indicates poor prognosis, and new lesion appearance means failure of treatment. Hence, besides DNA damage, exploring other bypasses might help to understand the mechanisms more comprehensively. Recently, it is reported that epithelial to mesenchymal transition (EMT), which initiates metastasis, accompanies with oxaliplatin resistance^1,2^, suggesting the two biological processes may share some common upstream signaling.

Whether during metastasis or under chemotherapeutics, cancer cells could develop several strategies against various stresses^3,4^. To cope with stress-induced RNA degradation, stress granules (SGs) are assembled to form dense globules, which help with storing stalled translation pre-initiation complexes in the cytosol^4–7^. Recently, ataxin-2-like (ATXN2L) was discovered as a novel regulator of SG^6^. It was reported that ATXN2L was widely expressed in immortalized cell lines, and ATXN2L-JAK2 fusion was found in CD4-positive T-cell lymphoma^8^. ATXN2L is a paralog of Ataxin-2 (ATXN2) but without abnormal polyQ expended track, which is conserved in most of the ATXNs and drives the pathogenesis of neurodegeneration. This suggests that they might share some especial characteristics. ATXN2 is now considered as a protein implicated in the neurodegenerative disorder diseases and associated with epidermal growth factor receptor (EGFR) signaling^9^. It is already known that EGFR signaling activation contributes to intrinsic oxaliplatin resistance^10,11^, while anti-EGFR treatment can reverse acquired oxaliplatin resistance^12^. However, apart from these limited clues, the function of ATXN2L in cancer remained greatly unknown. Whether ATXN2L is associated with oxaliplatin resistance or EGFR signaling was unclear.

Considering the close relationships between SG and cancer development^5,7^, we hypothesized that ATXN2L might participate in stress-related cancer malignant activities, which probably implies chemoresistance and EGFR signaling.

## Results

### ATXN2L upregulation in GC indicates adverse prognosis

**Fig. 1:**
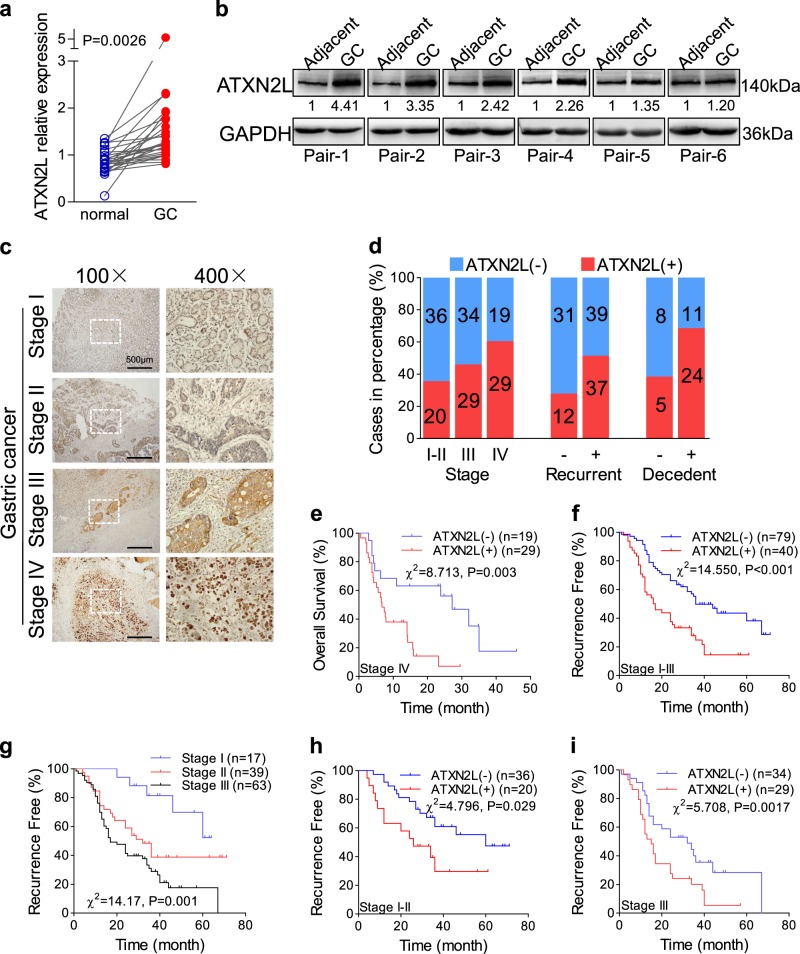
Ataxin-2-like (ATXN2L) upregulation in gastric cancer (GC) indicates adverse prognosis. **a** Relative expressions of ATXN2L mRNA in 27 pairs of GC tissues and adjacent noncancerous tissues from The Cancer Genome Atlas dataset. **b** ATXN2L protein expressions by Western blot analysis in fresh GC tissues and paired noncancerous tissues. **c** Representative immunohistochemical staining of ATXN2L in different TNM stage GC tissues. **d** The frequency of high and low ATXN2L expressions in GC patients when categorized by TNM stages, recurrent patients of stage I–III and decedent of stage IV. The number of patients in each stage was noted in the bars. Higher ATXN2L level indicates **e** shorter overall survival in stage IV patients, as well as **f** shorter recurrence-free survival in stage I–III patients. **g** Kaplan-Meier analysis of stage I–III patient recurrence-free survival time by different TNM stages. Higher ATXN2L level indicates shorter recurrence-free survival in **h** stage I–II patients and **i** stage III patients

To find out the expression status of ATXN2L in GC, we analyzed GC data from The Cancer Genome Atlas dataset, which included 27 pairs of cancer and adjacent noncancerous tissue. Generally, ATXN2L was significantly overexpressed in GC tissue (Fig. 1a). This was also confirmed by protein levels in fresh tissues that most pairs demonstrated higher ATXN2L expression in GC than the adjacent noncancerous (Fig. 1b). To figure out the clinical significance of ATXN2L on GC, we followed 167 GC patients in our hospital, and immunohistochemical (IHC) staining on treatment-naive GC tissues was performed (Fig. 1c). Among them, 48 were stage IV advanced GC patients who received only palliative treatments, and 119 were stage I–III patients who received curative resection. The frequency of ATXN2L high expression increased along with progression of cancer stage. In stage I–III patients, the portion of ATXN2L high expression was higher in recurrent patients. In stage IV, ATXN2L high expression was found to be positively correlated with mortality (Fig. 1d). Kaplan-Meier survival analysis was performed. ATXN2L high expression indicated shorter overall survival (OS) in stage IV patients (Fig. 1e) and recurrence-free survival (RFS) in stage I–III patients (Fig. 1f). When categorized by tumor stages, RFS significantly decreased in the stage III GC comparing to the stage I or II (Fig. 1g). Given the stage-dependent diversity, we further analyzed ATXN2L influence on RFS in early stage (stage I–II) and middle stage (stage III) patients, respectively. After eliminating the stage interference, ATXN2L high expression GC still showed a significantly greater risk of recurrence within the same stage (Fig. 1h, i). These clinical data suggests that the influence of ATXN2L on GC might cover different stages.

### ATXN2L promotes GC cell invasiveness without influencing proliferation

**Fig. 2:**
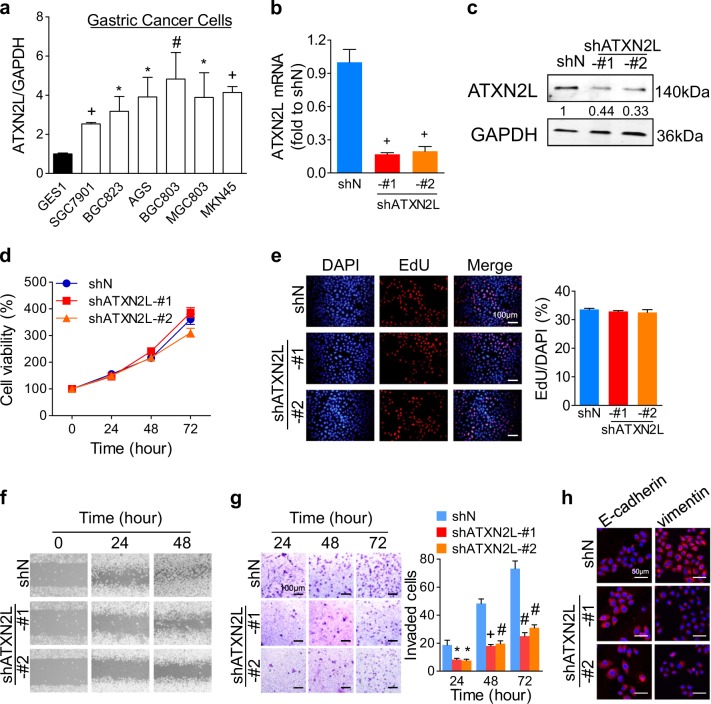
Ataxin-2-like (ATXN2L) promotes MGC803 cell migration and invasion without influencing proliferation. **a** ATXN2L mRNA levels in gastric cancer (GC) cell lines and human gastric mucosal cell line GES-1. ATXN2L **b** mRNA and **c** protein expression levels were successfully silenced by short hairpin RNAs (shRNAs). **d** Cell viability and **e** DNA synthesis ability of MGC803 cells were detected by methyl-thiazolyl-tetrazolium assay and EDU assay, respectively. **f** MGC803 cell migration activity and **g** invasiveness were evaluated by wound healing assay and transwell assay, respectively. **h** Silencing ATXN2L resulted in E-cadherin upregulation and vimentin downregulation in MGC803. Error bar represent SEM of triplicate repeated assays. Compared by Student’s *T*-test, *P < 0.05, ^#^*P* < 0.01, and ^+^*P* < 0.001

Before biological function exploration, ATXN2L expression level was detected in different cell lines. Compared to that in human gastric mucosal cell line GES-1, mRNA levels of ATXN2L were higher in most GC cell lines (Fig. 2a). MGC803 and MKN45 cell lines were used for subsequent experiments. Two short hairpin RNA (shRNA) that targeting ATXN2L were inducted into MGC803 respectively (Fig. 2b, c).

Unexpectedly, silencing ATXN2L did not have an impact on MGC803 and MKN45 cell viability (Fig. 2d and supplementary Figure S1a) or DNA synthesis (Fig. 2e and supplementary Figure S1b). However, silencing ATXN2L did slow down cell migration (Fig. 2f and supplementary Figure S1c) and inhibit cell invasion (Fig. 2g and supplementary Figure S1d). In protein level, silencing ATXN2L upregulated epithelial marker E-cadherin and downregulated mesenchymal phenotype marker vimentin expressions (Fig. 2h). These suggested ATXN2L might contribute to GC metastasis other than growth.

### ATXN2L upregulation contributes to intrinsic oxaliplatin resistance

**Fig. 3:**
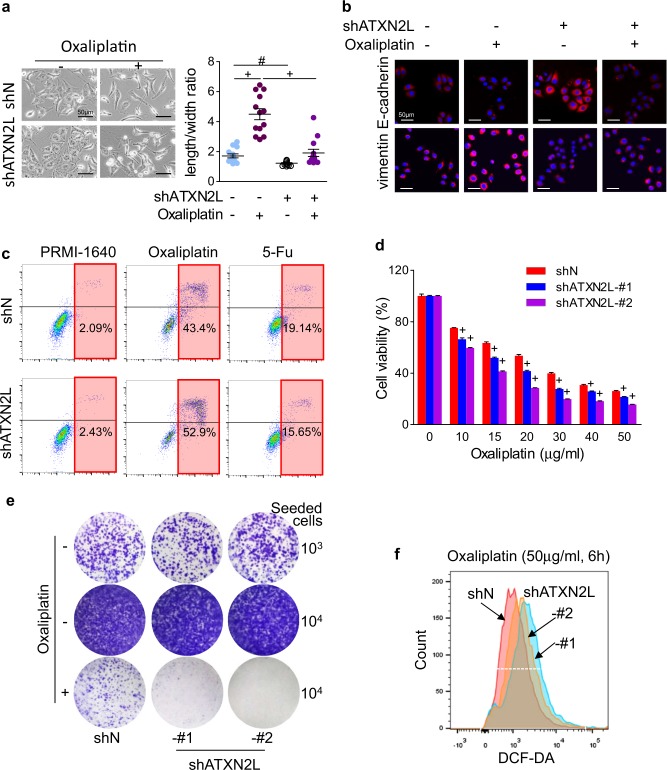
Ataxin-2-like (ATXN2L) upregulation contributes to intrinsic oxaliplatin resistance. **a** Oxaliplatin-induced MGC803 cell scattering and morphologic changes were suppressed by silencing ATXN2L. **b** Silencing ATXN2L reversed oxaliplatin-induced epithelial mesenchymal transition in MGC803 cells. **c** Apoptosis of shATXN2L and corresponding control MGC803 cells after oxaliplatin and 5-fluorouracil (5-Fu) treatment detected by flow cytometry. After oxaliplatin treatment, **d** survival rate (*n* = 4), **e** colony formation ability, and **f** reactive oxygen species production of shATXN2L and corresponding control MGC803 cells were detected. Error bar represent SEM of repeated assays. Compared by Student’s *T*-test, ^#^*P* < 0.01, and ^+^*P* < 0.001

It was reported that low-dose oxaliplatin could induce EMT in liver and colon cancers^1,13^. It was also found in MGC803 cells that 10 µg/ml oxaliplatin treatment prolonged cells to spindle shape and induced scattering. However, silencing ATXN2L depolarized the cells and maintained cluster formation (Fig. 3a), which exerted suppressive effect on oxaliplatin-induced EMT phenotype alteration (Fig. 3b). Since oxaliplatin is a first-line chemotherapeutic agent for GC, we wanted to know whether ATXN2L expression affects the therapeutic effect. Without external stress, knocking down ATXN2L did not significantly increase the apoptosis rate. Whereas, by adding oxaliplatin into culture median, much more apoptotic cells were detected in ATXN2L-silencing group. By contrast, silencing ATXN2L did not influence cell apoptosis when adding 5-fluorouracil, another commonly used GC chemotherapeutic agent (Fig. 3c). This suggested that ATXN2L was responsible for oxaliplatin-specific chemoresistance. It was also verified by methyl-thiazolyl-tetrazolium (MTT) assay performed with oxaliplatin concentration gradient that silencing ATXN2L greatly decreased MGC803 cell viability (Fig. 3d). As the result of intrinsic chemoresistance, cell colony formation of MGC803 was significantly inhibited by silencing ATXN2L under oxaliplatin condition (Fig. 3e). It was recently reported that redox homeostasis disorders contributed to oxaliplatin resistance, while excessive oxidative stress made cancer cell more vulnerable to oxaliplatin-induced apoptosis^14^. Hence, as an indicator of oxidative stress, reactive oxygen species (ROS) was analyzed in this study. As a result, ROS induction by oxaliplatin was more efficient when ATXN2L was knocked down (Fig. 3f), suggesting that ATXN2L was critical in oxidative stress modulation.

### Stress induced by oxaliplatin promotes ATXN2L expression and SG assembly

**Fig. 4:**
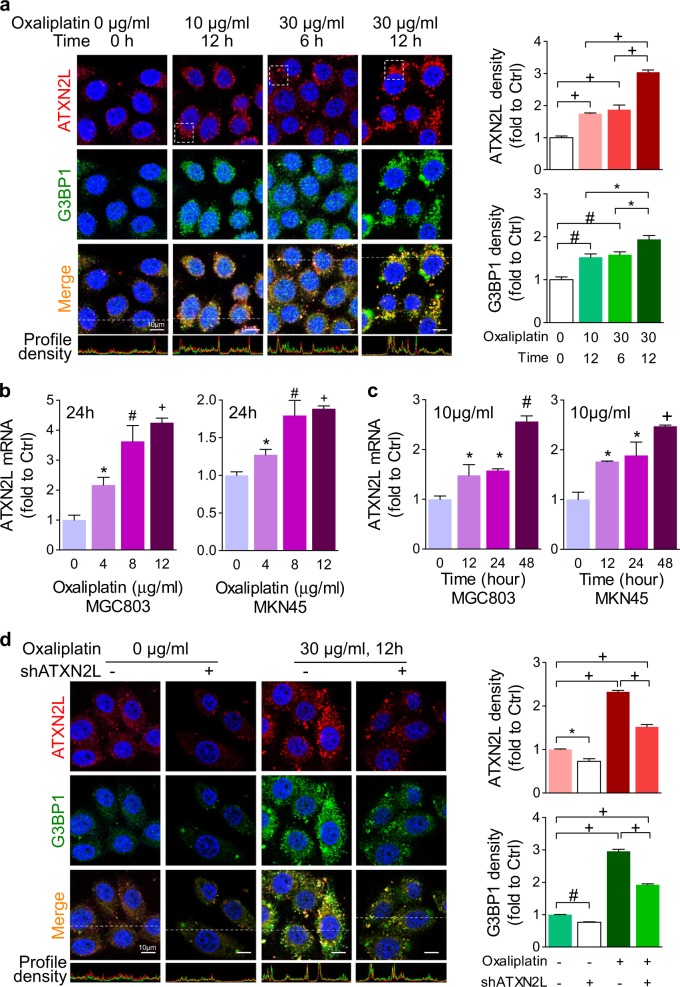
Stress by oxaliplatin promotes ataxin-2-like (ATXN2L) expression and stress granule (SG) assembly. **a** Under different concentrations and durations of oxaliplatin stimulation, the immunfluorescence staining expressions of ATXN2L and G3BP1 were enhanced by different levels, and ATXN2L coexpressed with G3BP1 in SG. **b** Concentration gradient and **c** time course of oxaliplatin treatments induced ATXN2L mRNA upregulation in MGC803 and MKN45 cells. **d** Silencing ATXN2L partly reversed the SG formation stimulated by oxaliplatin. Error bars represent SEM of triplicate repeated assays. Compared by Student’s *T*-test, *P < 0.05, ^#^*P* < 0.01, and ^+^*P* < 0.001

SG can be formed under oxidative stress, which was reported to facilitate cancer cells to acquire chemoresistance^5,15^. Therefore, we wanted to know how ATXN2L-related SG was assembled under oxaliplatin stress. GC cells were cultured in time course and concentration gradient. Stained by immunofluorescence antibodies, ATXN2L was found co-localized with SG marker Ras GTPase-activating protein-binding protein 1 (G3BP1) in cytoplasm. As indicated by G3BP1 and ATXN2L, few SGs were found in cytoplasm in the oxaliplatin-untreated control group. Yet, when oxaliplatin was added in, ATXN2L and G3BP1 protein expression was significantly increased time or concentration dependently. As a result, SG assembly was greatly enhanced, which was presented as ATXN2L and G3BP1 double-positive dense foci (Fig. 4a). Next, quantitative real-time PCR was performed to see whether ATXN2L mRNA levels were altered by oxaliplatin treatment. As expected, in both MGC803 and MKN45 cells, ATXN2L mRNA levels were upregulated gradually by oxaliplatin dose escalation (Fig. 4b). Similarly, ATXN2L mRNA levels also increased by culture time in these two cell lines (Fig. 4c). Next, SG was detected after knocking down ATXN2L. Silencing ATXN2L significantly inhibited G3BP1 expression. Moreover, SG assembly enhanced by oxaliplatin was inhibited by ATXN2L silencing (Fig. 4d). Here, silencing ATXN2L could not eliminate it completely. This was probably because that some other mechanisms or bypaths might get activated under such stress. Even so, it could be determined that ATXN2L was one of the important molecules coping with stress, which gave an explanation to why silencing ATXN2L enhanced the effects of oxaliplatin. The results also drew our interest to whether long-term oxaliplatin treatment could accumulate ATXN2L thus lead to acquired oxaliplatin resistance.

### Silencing ATXN2L expression reverses acquired oxaliplatin resistance

**Fig. 5:**
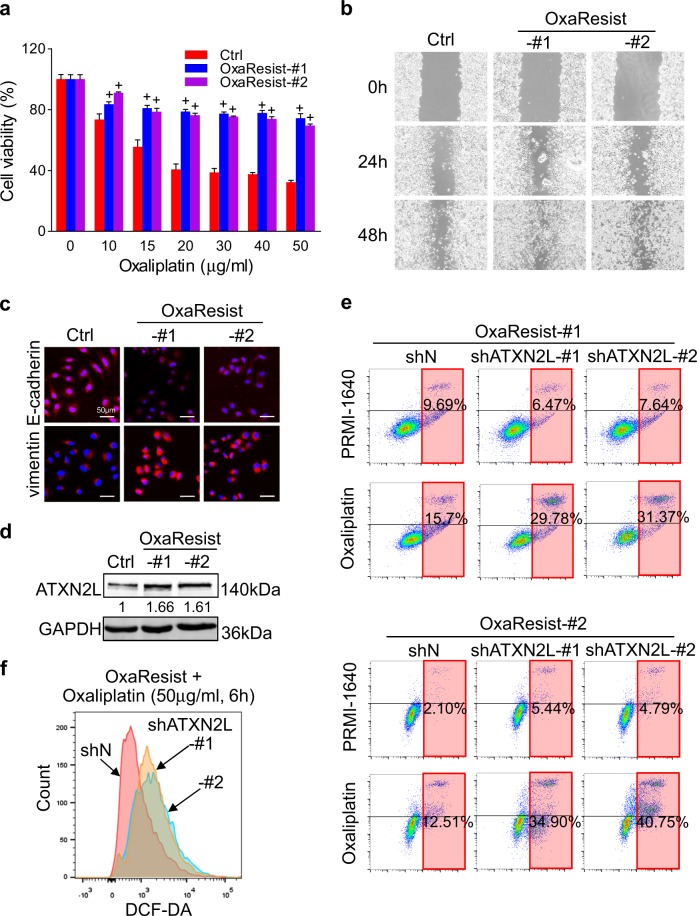
Silencing ataxin-2-like (ATXN2L) expression reverses acquired oxaliplatin resistance. **a** Two oxaliplatin-resistant MGC803 strains (OxaResist-#1 and -#2) were successfully induced and manifested by methyl-thiazolyl-tetrazolium assay under oxaliplatin stimulation of concentration gradient (*n* = 4). **b** Measured by wound healing assay, OxaResist strains showed enhanced cell migration activity. **c** Stable oxaliplatin resistance acquisition accompanied with epithelial mesenchymal transition. **d** ATXN2L protein expressions were significantly and stably upregulated in OxaResist strains. **e** Silencing ATXN2L expression increased apoptosis rates in OxaResist cells. **f** Reactive oxygen species production of OxaResist cells was greatly increased by ATXN2L knocking down. Error bar represent SEM of repeated assays. Compared by Student’s *T*-test, ^+^*P* < 0.001

Through 12 months’ concentration gradient induction, two strains of acquired oxaliplatin-resistant MGC803 cells (OxaResist-#1 and -#2) were successfully generated. Even under 50 μg/ml oxaliplatin, both OxaResist-#1 and -#2 still maintained over 70% viability, which was more than two folds compared to the untreated control cells (Fig. 5a). Cell motility was detected by wound healing assay, in which OxaResist cells showed higher migration ability (Fig. 5b). In protein level, OxaResist cells demonstrated mesenchymal-like phenotype with elevated vimentin and decreased E-cadherin expression (Fig. 5c). After long-term oxaliplatin induction, ATXN2L protein was stably upregulated in OxaResist cells (Fig. 5d). To explore whether ATXN2L influences the function of OxaResist cells, we used the two shRNA (shATXN2L-#1 and -#2) to knock down ATXN2L expression (Fig. 5e). As a result, whether silencing ATXN2L or not, the apoptosis rates of OxaResist cells kept at low level without oxaliplatin treatment. Whereas, after cultured under 30 μg/ml oxaliplatin for 24 h, silencing ATXN2L greatly enhanced apoptosis rates of OxaResist cells (Fig. 5e). Indicated by ROS, oxidative stress was significantly increased by shATXN2L induction (Fig. 5f). These results demonstrate that silencing ATXN2L expression could reverse acquired oxaliplatin resistance.

### EGF promotes ATXN2L expression via PI3K/Akt signaling activation

**Fig. 6:**
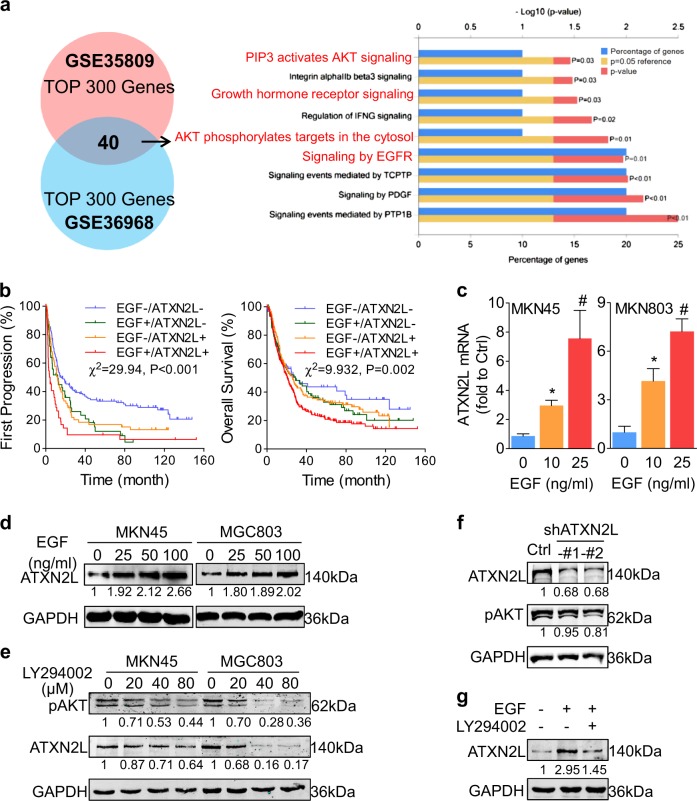
Epidermal growth factor (EGF) promotes ataxin-2-like (ATXN2L) expression via PI3K/Akt signaling activation. **a** Bioinformatic analysis of datasets GSE35809 and GSE36968 showed that ATXN2L correlated proteins might probably enriched in EGF receptor and PI3K/Akt signaling. **b** Progression-free survival and overall survival were analyzed based on EGF and ATXN2L expression using multiple gastric cancer datasets, including GSE14210 (*n* = 146), GSE15459 (*n* = 200), GSE22377 (*n* = 43), GSE29272 (*n* = 268), and GSE51105 (*n* = 94). Gradient concentration of EGF treatment upregulated the **c** mRNA (*n* = 3) and **d** protein expressions of ATXN2L in MKN45 and MGC803 cells. **e** Gradient concentration of LY294002 treatment induced Akt dephosphorylation and ATXN2L downregulation in MKN45 and MGC803 cells. **f** Silencing ATXN2L did not influence Akt phosphorylation level greatly in MGC803 cell. **g** EGF-upregulated ATXN2L expression was reversed by LY294002 for Akt signaling blockade. Error bar represent SEM of repeated assays. Compared by Student’s *T*-test, *P < 0.05, and ^#^*P* < 0.01

To find out ATXN2L-related signaling, we performed bioinformatics analysis. Two datasets, GSE36968 and GSE35809, which included primary GC tissue RNA profiling from Asian and Austrian populations respectively, were analyzed by the Search-based Exploration of Expression Compendium (SEEK) (http://seek.princeton.edu/). Top 300 ATXN2L correlated genes were selected from either dataset, and the intersection of 40 genes was taken for Functional Enrichment Analysis Tool (FunRich) (http://www.funrich.org/) analysis. Through biological pathways enrichment, we found that most of the correlated genes lay in EGFR and Akt signaling (Fig. 6a). Thus, we performed GC survival analysis using online Kaplan-Meier plotter (www.kmplot.com) to explore the role of EGF and ATXN2L in clinical outcomes. GC survival datasets GSE14210, GSE15459, GSE22377, GSE29272, and GSE51105 were included. As a result, patients with low levels of both EGF and ATXN2L demonstrated the best outcomes concerning the time course until first progression and OS. On the contrary, patients with high levels of both EGF and ATXN2L have the poorest outcomes (Fig. 6b). The results suggest that ATXN2L might have a close correlation with EGF-related signaling.

To verify our hypothesis, different concentrations of recombinant human EGF were added to stimulate MKN45 and MGC803 cells. As anticipated, ATXN2L mRNA (Fig. 6c) and protein (Fig. 6d) expressions were upregulated in a dose-dependent manner in both cell lines. It is already known that PI3K/Akt signaling activation is one of the downstream effects of EGF stimulation. We then used LY294002, a broad-spectrum inhibitor of PI3K, to block Akt phosphorylation. Along with Akt dephosphorylation, ATXN2L expression was reduced dose dependently in MKN45 and MGC803 lines (Fig. 6e). Also, silencing ATXN2L by shRNA did not affect Akt phosphorylation level (Fig. 6f), suggesting that ATXN2L was the downstream target of PI3K/Akt signaling. Moreover, by adding LY294002 under the condition of EGF administration, EGF-upregulated ATXN2L expression was dramatically reversed (Fig. 6g), suggesting that EGF enhanced ATXN2L expression through PI3K/Akt signaling activation.

### EGFR/ATXN2L signaling is involved in GC oxaliplatin resistance and invasiveness

**Fig. 7:**
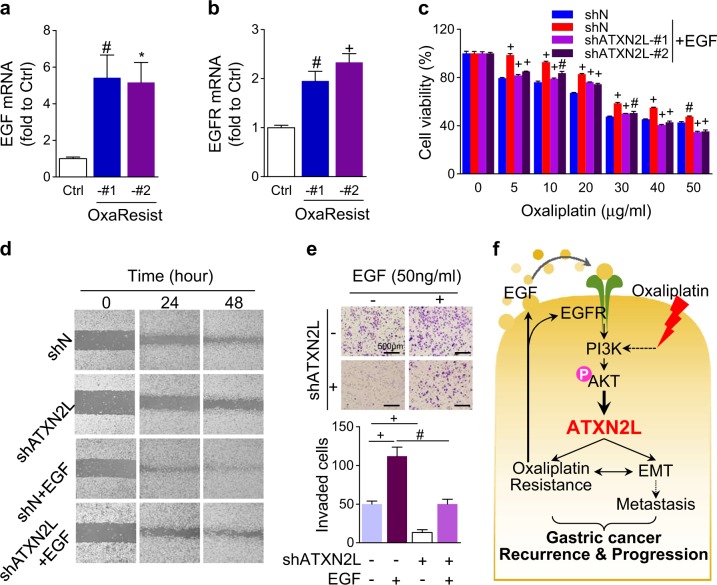
EGFR/ATXN2L signaling is involved in gastric cancer (GC) cell oxaliplatin resistance and invasiveness. OxaResist strains showed upregulated **a** epidermal growth factor (EGF) and **b** EGF receptor (EGFR) mRNA expressions (*n* = 3). **c** EGF increased the chemoresistance to oxaliplatin in MGC803 cells, while silencing the downstream ataxin-2-like (ATXN2L) re-sensitize cells to oxaliplatin even under EGF stimulation (*n* = 4). **d** Migration and **e** invasion (*n* = 3) activities of MGC803 cells were greatly enhanced by EGF but reversed by ATXN2L silencing. **f** Schematic graph of current study. ATXN2L expression can be upregulated by EGF via PI3K/Akt signaling. ATXN2L upregulation induces epithelial mesenchymal transition and intrinsic and acquired oxaliplatin resistance, which consequently leads to GC recurrence and progression. Oxaliplatin treatment can induce ATXN2L upregulation, while stable oxaliplatin-resistant strains shows increased ATXN2L as well as EGF and EGFR expressions. These results draw a positive feedback loop connecting EGF, ATXN2L, and oxaliplatin resistance. Full lines show the verified relationships in current study, while imaginary lines show the documented conclusions by other studies. Error bar represent SEM of repeated assays. Compared by Student’s *T*-test, *P < 0.05, ^#^*P* < 0.01, and ^+^*P* < 0.001

Interestingly, we found that OxaResist cells also manifested elevated mRNA expressions of EGF (Fig. 7a) and EGFR (Fig. 7b). The impact of EGF and ATXN2L on oxaliplatin resistance was assessed in MGC803 cells. In oxaliplatin-sensitive cells, recombinant human EGF induction greatly maintained cell viability under different concentration oxaliplatin treatments (Fig. 7c). However, silencing the downstream ATXN2L re-sensitized cells to oxaliplatin even under EGF stimulation (Fig. 7c). These results suggest that GC oxaliplatin resistance could be acquired by EGFR/ATXN2L signaling. Meanwhile, we also analyzed the effect of EGFR/ATXN2L signaling on cell migration and invasion. In the wound healing assay, EGF significantly accelerated MGC803 migration, while silencing ATXN2L under EGF condition potently held back the scratch recovery (Fig. 7d). In the transwell assay, EGF-promoted cell invasiveness was also augmented by ATXN2L inhibition (Fig. 7e). These results show that ATXN2L upregulation was necessary for EGF-induced GC migration and invasion.

## Discussion

In this study, we discovered the novel functions of ATXN2L in cancer. ATXN2L contributed to GC recurrence and progression even under the treatment of oxaliplatin. EGF and its downstream PI3K/Akt signaling activation promoted ATXN2L expression. On one hand, ATXN2L upregulation promoted migration and invasion via EMT. On the other hand, ATXN2L helped SG assembly under oxaliplatin-induced stress, and ATXN2L high expression led to intrinsic and acquired oxaliplatin resistance. In turn, oxaliplatin-resistant cell lines demonstrated increased ATXN2L as well as EGF and EGFR expressions. Given that oxaliplatin was previously reported to activate PI3K/Akt signaling in a compensatory way, these results built a positive feedback loop connecting EGF, ATXN2L, and oxaliplatin resistance. To break up the loop, we showed that silencing ATXN2L could reverse the intrinsic and acquired oxaliplatin resistance (Fig. 7f). This study suggests that ATXN2L may serve as a potential prognostic marker and therapeutic target in GC, especially when considering using oxaliplatin-based chemotherapy.

Current study is the first one that unveiled the prognostic significance of ATXN2L expression in cancer, which was then identified as invasion promotion and chemoresistance acquisition in our cellular biological experiments. There is a mutual promoting relationship between oxaliplatin resistance and EMT. EMT induction was showed to promote oxaliplatin resistance acquisition^16^, while blocking EMT-associated signatures sensitized cancer cells to oxaliplatin^17^. Reciprocally, chronic oxaliplatin resistance could also induce EMT in cancer cells^18^. Here we proved that oxaliplatin treatment upregulated ATXN2L and converted epithelial-like GC cells to mesenchymal-like ones to escape stress. Moreover, we also proved that ATXN2L was necessary for SG assembly under oxaliplatin treatment, which was another compensative process accommodating cancer cells to critical stresses. However, it is interesting that ATXN2L had no effect on cell proliferation. From the evolutionary perspective, ATXN2L is a stress response molecule. Cancer cells were driven to a dormant state by stresses, and keeping a stable or even relatively lower proliferative rate is a self-protection manner. Moreover, SG induced by ATXN2L could store RNA, which stalled protein translation. By contrast to proliferation, other biological process not involving cell division, for example invasion, calls for much less protein synthesis. By this, it is not difficult to understand why ATXN2L promotes cell movement without affecting proliferation.

Unlike oxaliplatin, silencing ATXN2L did not enhance 5-fluorouracil toxicity. Due to the principal pharmacological differences, where oxaliplatin forms crosslinks in DNA and 5-fluorouracil inhibits thymidylate synthase, the two chemo agents are usually used in a combination to avoid cross chemoresistance. Hence, it is acceptable that silencing ATXN2L could not cover 5-fluorouracil. However, the detailed mechanisms of ATXN2L specificity to oxaliplatin resistance need further explorations.

As to transcriptional modulation, we discovered a positive feedback loop connecting EGFR/PI3K/Akt signaling, ATXN2L, and oxaliplatin resistance. Although cetuximab, an anti-EGFR monoclonal antibody, failed to achieve satisfactory outcomes in GC, it was reported that cells with EGFR signaling activation were less responsive to oxaliplatin^19^, while blocking EGFR enhanced the effect of oxaliplatin on GC cell lines^11^. This also suggests there might be some other mechanisms influencing anti-EGFR effect. In addition to ATXN2L upregulation, we also demonstrated that acquired oxaliplatin-resistant cell lines had elevated EGF and EGFR expression. Since oxaliplatin could induce PI3K/Akt signaling activation via Notch-1 signaling^20^, Akt-induced ATXN2L upregulation might be the key event for oxaliplatin resistance acquisition. In other words, silencing ATXN2L might be a promising strategy against EGFR signaling-associated chemoresistance.

## Materials and methods

### Patients and tissue histological analysis

The clinical study was approved by the Nanfang Hospital Ethics Review Board. The patients were histologically diagnosed with GC at Nanfang Hospital, Southern Medical University (Guangzhou, China). Therein, 119 patients were stage I–III patients who then received curative resection, while the other 48 stage IV patients lost curative opportunity and received only palliative treatments. Tumors were staged according to the *AJCC Cancer Staging Manual* (8th edition, 2018). IHC staining of ATXN2L expression was analyzed by a semiquantitative method as previously described^3^. The intensity of staining was scored as 0 (negative), 1 (weak), 2 (medium), or 3 (strong), while the extent of staining was scored as 0 (0% of cells stained), 1 (1–25%), 2 (26–50%), 3 (51–75%), or 4 (76–100%). The final protein expression score was calculated by the product of intensity and extent scores. The experiments were undertaken with the consent of each subject, and the study methodologies conformed to the standards set by the Declaration of Helsinki.

### Cell culture

Cancer cell lines of SGC7901, BGC823, MGC803, MGC803, and MKN45, as well as gastric mucosal cell line GES-1, were routinely cultured in complete RPMI-1640 medium (Invitrogen, CA, USA) with 10% fetal bovine serum at 37 °C under 5% CO_2_. Oxaliplatin (Sigma-Aldrich, Saint Louis, MO, US) and 5-fluorouracil (Sigma-Aldrich) were added into culture medium up to indicated concentration, and cells were cultured for indicated period as described in the Results section. To induce OxaResist lines, MGC803 cells were cultured in complete RPMI-1640 medium with progressively increased oxaliplatin, which was of initial concentration 5 μM to 50 μg/ml finally for more than 1 years. Recombinant human EGF (R & D system, Minneapolis, MN, US) was added into culture medium as intended. Inhibitor of LY2940026 (5 μM) was used to block PI3K activation.

### Western blot

For Western blot, antibodies of ATXN2L (Proteintech, Rosemont, IL), pAKT (Cell Signaling Tech., Danvers, MA, US), and GAPDH (Proteintech) were used. Procedures was as previously described^21^. Generally, cells or tissues were solubilized in lysis buffer (KeyGEN, Nanjing, China), and total protein concentration was determined by BioRad BCA method (Pierce, Rockford, IL, USA). After electrophoresed on 10% sodium dodecyl sulfate-polyacrylamide gels, protein was electroblotted onto polyvinylidene fluoride membranes. Then, membranes were blocked with 5% bovine serum albumin and incubated overnight at 4 °C with antibodies against ATXN2L, pAKT, or GAPDH, respectively. After incubated by secondary antibodies, blots were visualized using enhanced chemiluminescence.

### Immunofluorescence

For immunofluorescence, rabbit antibodies of ATXN2L (Sigma-Aldrich), E-cadherin (Cell Signaling), and vimentin (Cell Signaling), and mouse antibody G3BP1 (Abcam) were used. Immunofluorescence staining was performed as previously described^3^. Cells were seeded onto glass-bottom dish and fixed by polyformaldehyde. Cellular membrane was permeabilized by 0.2% Triton X-100/phosphate-buffered saline (PBS) solution. After blocked by 0.5% Tween-20/PBS for 1 h, dish was incubated with antibodies against ATXN2L, E-cadherin, vimentin, or G3BP1 for 12 h. Then cells were incubated by secondary antibodies, and nucleus were stained by 4′,6-diamidino-2-phenylindole. Cells were observed by Olympus FV10i Olympus laser confocal system (Olympus, Tokyo, Japan), and analyzed by FV10-ASW 3.0 Viewer (Olympus). Staining intensity were half quantified using Image-Pro Plus 6.0 (Media Cybernetics, Bethesda, MA, USA).

### Quantitative real-time PCR

The Trizol kit (Life Science, Carlsbad, CA, USA) for cellular total RNA extraction and a reverse transcriptase (Roche, Penzberg, Germany) were used according to the protocols as recommended. Quantitative real-time PCR was performed using the SYBR Green I Master kit (Roche) on a LightCycler 480 system. Primer sequences involved in the present study are as follows: ATXN2L-F CGCAGCAACACCAGGAGA, ATXN2L-R GCAGCATTCTGGAATTGTTGTA; EGF-F TGTCCACGCAATGTGTCTGAA, EGF-R CATTATCGGGTGAGGAACAACC; EGFR-F TTGCGCAAAGTGTGTAACG, EGFR-R GTCACCCCTAAATGCCACCG; and GAPDH-F AGGGCTGCTTTTAACTCTGGT, GAPDH-R CCCCACTTGATTTTGGAGGGA.

### ROS detection and cell apoptosis assays

After oxaliplatin treatment, ROS was stained with dichlorofluorescin diacetate (DCFH-DA) dye (Jiancheng, Nanjing, China). Viable cells were incubated with 10 μM DCFH-DA for 20 min, 37 °C. Cell apoptosis was stained by Annexin V-fluoroscein isothiocyanate (Beyotime Biotechnology, Haimen, China) for 15 min, 37 °C, and then propidium iodide 5 min, 4 °C. Then, cells were washed by serum-free medium for three times before using the LSRFortessa system (BD Biosciences, San José, CA, USA) for flow cytometry detection^3^.

### Cell viability, proliferation, and colony formation assays

Cells viability was measured by MTT assay as previously described^22^. Cell proliferation ability was reflected by EdU incorporation assay using the EdU Assay Kits (Life Technologies, Carlsbad, CA, USA) according to the manufacturer’s instruction and observed under fluorescent microscope. Colony formation was performed using soft agar. Trypsinized cells were suspended in 2 ml of complete medium with 0.3% agar and plated onto a bottom layer with 1% agar (Sigma, St. Louis, MO, US) in complete medium. After 14 days culturing, cells were observed by crystal violet.

### Migration and invasion

Cell migration was detected by wound healing assay. After seeded onto a 12-well plate at a density of 5 × 10^4^/well, cells were cultured till 90% confluence. The monolayer was scratched using a 10-µl pipette tip. Cells were cultured in complete medium, 37 °C. Wound closure was observed under microscope. Cell invasiveness was evaluated by Transwell assay. Cells were planted in serum-free medium in the upper chamber (Dow Corning, Corning, NY, US), which consisted of 8μm membrane filter inserts (1 × 10^5^/ml, 200 ml/well) with Matrigel. The lower chamber was complete medium with serum as chemoattractant. After 48 h culturing, cells at upper chamber were stained by crystal violet and photographed under microscope.

### Statistical analysis

Data were analyzed using the SPSS19 software. The results were presented as the mean ± SEM. For means comparison, experiments were repeated at least thrice, and the significance of difference was analyzed using one-way analysis of variance or Student’s *T*-test. Survival rates were analyzed according to the Kaplan-Meier method and examined by the log-rank test. *P* < 0.05 were considered statically significant.

## Supplementary information

supplementary Figure
