# Supplementary material for: ATXN2L upregulated by epidermal growth factor promotes gastric cancer cell invasiveness and oxaliplatin resistance
Source: Cell Death Dis. 2019 Feb 20;10(3):173. doi: 10.1038/s41419-019-1362-2 (PMC6382779; doi:10.1038/s41419-019-1362-2)
Supplement: Supplementary file 1 — supplementary Figure [file 41419_2019_1362_MOESM1_ESM.pdf]

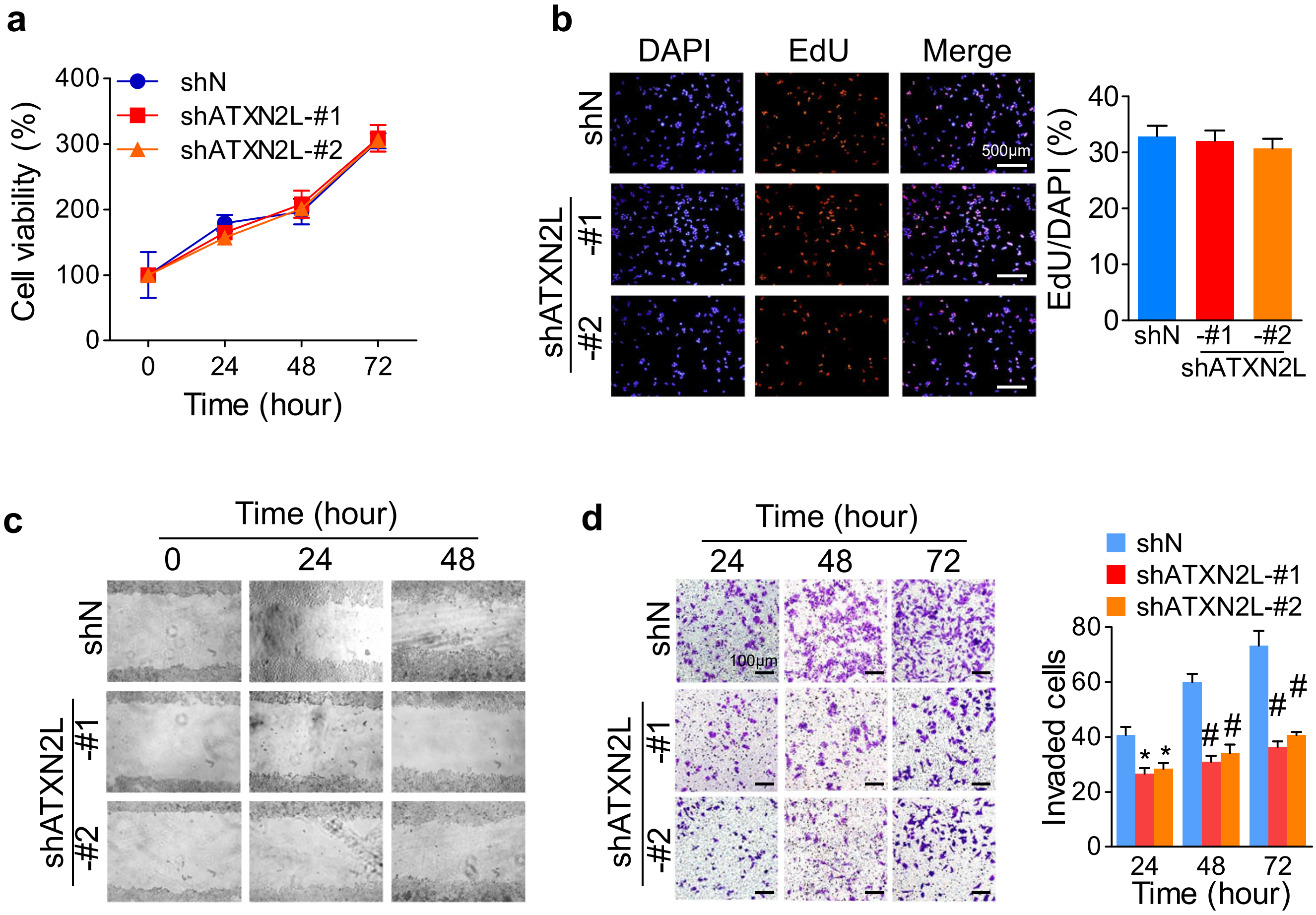

Supplementary Figure S1

ATXN2L promotes MKN45 cell migration and invasion without influencing proliferation.

(a) Cell viability and **(b)** DNA synthesis ability of MKN45 cells were detected by MTT assay and EDU assay, respectively. **(c)** MKN45 cell migration activity and **(d)** invasiveness were evaluated by wound healing assay and transwell assay, respectively. Error bar represent SEM of triplicate repeated assays. Compared by Student's T-test, **P*<0.05 and #*P*<0.01.
